# Molecular Mechanisms and Biological Functions of Autophagy for Genetics of Hearing Impairment

**DOI:** 10.3390/genes11111331

**Published:** 2020-11-11

**Authors:** Ken Hayashi, Yuna Suzuki, Chisato Fujimoto, Sho Kanzaki

**Affiliations:** 1 Department of Otolaryngology, Kamio Memorial Hospital, Tokyo 101-0063, Japan; 2 Department of Biochemistry, Nihon University, Tokyo 173-0032, Japan; meyu18003@g.nihon-u.ac.jp; 3 Department of Otolaryngology-Head and Neck Surgery, Keio University, Tokyo 160-8582, Japan; skan@keio.jp; 4 Department of Otolaryngology-Head and Neck Surgery, The University of Tokyo, Tokyo 113-8655, Japan; cfujimoto-tky@umin.ac.jp

**Keywords:** classical degradative autophagy, genetics of hearing impairment, autophagy- and lysosomal function-related genes, congenital disorder

## Abstract

The etiology of hearing impairment following cochlear damage can be caused by many factors, including congenital or acquired onset, ototoxic drugs, noise exposure, and aging. Regardless of the many different etiologies, a common pathologic change is auditory cell death. It may be difficult to explain hearing impairment only from the aspect of cell death including apoptosis, necrosis, or necroptosis because the level of hearing loss varies widely. Therefore, we focused on autophagy as an intracellular phenomenon functionally competing with cell death. Autophagy is a dynamic lysosomal degradation and recycling system in the eukaryotic cell, mandatory for controlling the balance between cell survival and cell death induced by cellular stress, and maintaining homeostasis of postmitotic cells, including hair cells (HCs) and spiral ganglion neurons (SGNs) in the inner ear. Autophagy is considered a candidate for the auditory cell fate decision factor, whereas autophagy deficiency could be one of major causes of hearing impairment. In this paper, we review the molecular mechanisms and biologic functions of autophagy in the auditory system and discuss the latest research concerning autophagy-related genes and sensorineural hearing loss to gain insight into the role of autophagic mechanisms in inner-ear disorders.

## 1. Introduction

Cells are continuously exposed to various stresses, including both extracellular oxidative stress as well as intracellular endoplasmic reticulum (ER) stress. There is a positive feed-forward loop between oxidative stress and ER stress in cells. Oxidative stress occurs when the proper balance between antioxidants and reactive oxygen species (ROS) is lost. A higher production of ROS may change DNA structure, resulting in cell death, including apoptosis, necrosis, and necroptosis or cellular senescence. ER stress activates the signaling pathway of the unfolded protein response (UPR) triggered in response to the accumulation of unfolded or misfolded proteins in the ER. In cases where ER stress cannot be reversed, cellular functions deteriorate, often leading to cell death. Various stressors can disturb the intracellular redox balance, accumulating protein aggregation, and misfolding or unfolding proteins, leading to conformational disease. Inner-ear diseases may have aspects of conformational disease. Based on this concept, considerable research has been conducted on apoptosis and antioxidants in inner-ear diseases [1,2,3,4,5,6]. However, in the case of patients with hearing loss, the hearing levels and patterns are often diverse and not permanent. As a result, it may be difficult to explain hearing loss only from the aspect of cell death, apoptosis, necroptosis, or necrosis at the cellular level. Therefore, we focused on autophagy as a cellular phenomenon functionally competing against cell death for auditory cell fate decision. The lysosomal degradation pathway of autophagy (referred to as macroautophagy) plays an important role in adaptation to cellular stress, clearance of autophagic cargo (damaged organelles, intracellular pathogens, or protein aggregates), cellular development and differentiation, and mitigation of genomic damage. Crosstalk occurs among apoptosis, necroptosis, and autophagy [7]. Since the autophagic process controls auditory cell fate, protecting against hearing impairment, autophagy-related genes could potentially hold the key to the genetics of hearing impairment. To the best of our knowledge, there is no review article describing the effects of the autophagy process on the genetics of hearing impairment and autophagy–lysosomal function-related genes for hearing impairment. In this article, the first part describes the mechanisms and biologic functions of autophagy as a decision factor in auditory cell fate and the role of autophagy in the auditory system (or hearing), while the second part focuses on the relationship between autophagy (elongation and completion steps)- and lysosomal function (fusion step)-related genes and hearing loss, congenital disorder of autophagy with hearing loss, and the effect of autophagy for genetics of hearing loss.

## 2. The Mechanisms and Biologic Functions of Autophagy

Autophagy plays fundamental roles in cellular homeostasis and exerts a major impact on cells as the fate decision factor under various physiological and pathologic conditions [8]. Since Professor Ohsumi won the Nobel Prize in 2016 for his seminal research on autophagy, many inner-ear researchers have placed autophagy as the central target of their research. Today, the relationship between autophagy and inner-ear disease is a hot spot in inner-ear research.

### 2.1. Autophagy Gene-Dependent Pathways for the Formation of Autophagosome

**Figure 1:**
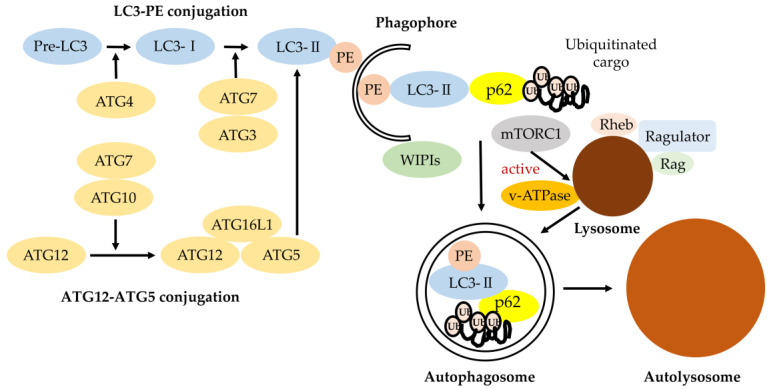
Classical degradative autophagy (macroautophagy). The autophagy-related gene (ATG) conjugation systems (LC3-PE and ATG12–ATG5) are important for degrading the inner autophagosomal membrane. Phagophore elongation involves two ubiquitin-like conjugation systems (LC3-PE and ATG12–ATG5 conjugation). ATG7 and ATG10 operate sequentially to catalyze the formation of ATG12–ATG5:ATG16L1 complexes. ATG4, ATG7, and ATG3 cooperate to cleave the precursors of LC3-like proteins into their mature forms, followed by conjugation to phosphatidylethanolamine (PE) and recruitment to autophagosomes forming with the support of WD-repeat protein interacting with phosphoinositide (WIPI) proteins. LC3 and LC3 homologs enable autophagosomes with the ability to bind autophagic substrates including p62 for ubiquitinated cargo [10]. The mammalian target of rapamycin (mTOR)C1 amino-acid-sensing pathway. V-ATPase triggers the guanine nucleotide exchange factor activity of Rag small GTP-binding protein in an amino-acid-dependent manner, which is followed by the recruitment of mTOR to lysosomal membranes. Upon its localization to the lysosome, mTORC1 kinase is activated by the small GTP-binding protein Rheb, which receives input from growth factor signaling. The lysosome is responsible for recycling amino acids and cellular components via degradation of proteins and other macromolecules, although acidification of the lysosomal lumen is dispensable for mTORC1 signaling.

As shown in Figure 1, classical degradative autophagy (macroautophagy) involves the delivery of cytoplasmic cargo to the lysosome for degradation. All autophagy-related genes (*ATGs*) are required for efficiently-sealed autophagosome formation and proceeding to fusion with lysosomes. The subsequent elongation and closure of the isolation membrane (phagophore) is mediated by two ubiquitin-like *ATG* conjugation systems, *ATG5–ATG12* and *LC3* (light chain 3)-PE, in mammals. These *ATG* conjugation systems are important for driving the biogenesis of the autophagosomal membrane [9].

The ubiquitin-like protein ATG12 is conjugated to ATG5 by ATG7. ATG16L1 and ATG12–ATG5 form a complex. This ATG16L1 complex specifically localizes to the isolation membrane (phagophore) and then dissociates from it for the completion of autophagosome formation. LC3 is processed at its C terminus by Atg4 and then becomes LC3-I. LC3-I is subsequently conjugated with phosphatidylethanolamine (PE) to become LC3-II by ATG7 (E1-like) and ATG3 (E2-like) and recruited to autophagosomes, forming with the support of WD-repeat protein interacting with phosphoinositide (WIPI) proteins. LC3-II enables autophagosomes to bind p62 for ubiquitinated cargo [10].

### 2.2. Autophagy Regulation by Lysosome through mTORC1 and v-ATPase

The inactivation of mTORC1 (mechanistic or mammalian target of rapamycin complex1) is one of the main inducers of autophagy. Multiple cues, including cellular amino acid levels or oxidative stress, modulate mTORC1 activity. Importantly, the recruitment to the lysosomal lumen and activation of mTORC1 requires lysosomes and vacuolar H^+^-ATPase (V-ATPase) (Figure 1). The V-ATPases are electrogenically-conserved proton pumps that acidify multiple intracellular organelles and extracellular compartments and are implicated as critical components of cellular signal transduction pathways including the wingless-related integration site (Wnt), Notch, and the mechanistic or mammalian target of rapamycin (mTOR) signaling. These three molecule-signaling cascades are linked to cell-growth regulation, coordinating downstream pathways involved in aging control. Due to these dependencies, complete loss of V-ATPase activity results in embryonic lethality in mammals. Partial loss is related to multiple disease states, including neurodegeneration or cancer [11]. The lysosome provides the key indication of cell metabolic state including autophagy, enhancing cellular clearance based on lysosomal mTOR-V-ATPase signaling [12,13].

## 3. The Role of Autophagy in Auditory System (or Hearing)

### 3.1. Otic Epithelium

A previous study [14] indicated that ATG5, beclin-1(ATG6), and LC3B (ATG8) are expressed during early development of the chicken inner ear, and that the otic epithelium has intense lysosomal activity and numerous autophagic vesicles, especially at neuroblast exit zones. Autophagy is an active process during early inner-ear development, providing the energy required for the generation of neuronal otic precursors via the clearing of dead neuroepithelial cells; autophagic activity is necessary for the otoconial biogenesis in inner-ear development.

### 3.2. Hair Cells, Spiral Ganglion Cells, and Brain Stem Nuclei

There were few reports on the relationship between autophagy and hearing loss before Professor Yoshinori Ohsumi’s Nobel Prize-winning work of 2016. Since this time, the number of reports has dramatically increased. Most inner-ear research has focused on the function of autophagy as constituting an important mechanism for the recycling of cytoplasmic materials and in fine cleaning and rejuvenating extranuclear compartments, especially in non-diving cells (or postmitotic cells) as typified by neurons [15]. In the auditory pathway, hair cells (HCs) in the cochlea convert sound information into electrical signals, then carry these signals to the central nervous system (CNS) via chemical synapses on the spiral ganglion (SG) neurons dendrites [16,17]. The central afferents of these SG neurons converge to form the auditory nerve, connecting to the cochlear nuclei in the brainstem [18].

A previous study [19] confirmed the expression of the autophagy machinery genes (*BECN1*, *ATG4g*, *ATG5,* and *ATG9a*) by qRT-PCR in the E18.5 mouse cochlea and the expression of *BECN1*, *ATG4g* and *ATG5* in the brain-stem nuclei. Autophagy was also confirmed to be abundant in spiral ganglion neurons by the expression of LC3B. The most important aspect of this study is that inner-ear autophagy flux was revealed to be developmentally regulated and is lower at perinatal stages than in the adult mouse. Another study [20] indicated that the deletion of *ATG5* results in the degeneration of hair cells (HCs) and profound congenital hearing loss. In this study, basal autophagy flux was detected in both the inner and outer hair cells, whereas autophagosome formation was suppressed in the *ATG5*-deficient HCs. Aggregates containing ubiquitin and p62 also accumulated. This suggests that *ATG5* deficiency results in congenital profound hearing loss due to the degeneration rather than maldevelopment of auditory HCs and that *ATG5* in cochlear HCs is essential for the maintenance of the morphology of these cells and acquiring normal hearing acuity. These in vivo studies suggested that autophagy plays a crucial role in the development, maintenance of morphology, and functional maturation of the auditory system, and that abnormality of the autophagy machinery genes may cause both congenital and acquired sensorineural hearing loss.

### 3.3. Synapse Ribbon

Glutamatergic ribbon-type synapses (cochlear ribbon synapses) are composed of molecular machinery transducing mechano-electric components on the apical side of inner hair cells (IHCs), connecting IHCs and spiral ganglion neurons (SGNs). Although ribbon synapses are immature at birth, they mature, morphologically and functionally, between IHCs and SGNs with hearing onset during development, coordinating with SGN (type 1) myelination, spontaneous activity, and synaptic pruning. A recent study [21] indicated that autophagy plays an essential role in the development and maturation of cochlear ribbon synapses in mice. According to this report, autophagy in IHCs was highly activated in the early stage of hearing development (P1 to P15) and then decreased at P28 to P30. In contrast, deficiency of autophagy before hearing onset impaired the pruning and refinement of ribbon synapses in IHCs and the impairment of autophagy flux results in the exocytosis of cochlear IHCs in postnatal mice. They proposed that in postnatal mice, the remodeling process of ribbon synapses in cochlear IHCs during development may be mainly controlled by autophagy, and that deficiency of autophagy at the early stage of hearing development may induce auditory disorders via impairment of cochlear ribbon synapses.

### 3.4. Auditory Neurons

The SGNs of the cochlea transmit all auditory information to the brain. In a recent study using single-cell RNA sequencing [22], four types of SG neurons, including three novel subclasses of type I neurons (Ia, Ib, and Ic neurons) and the type II neurons that exist at birth, were identified, and a comprehensive genetic framework that constructs their potential synaptic communication patterns was provided. The authors also found that many inhibitory modulators of *TGFβ* signaling (Smad6, Smad7, Nog, Nbl1, Smad9, and Smurf2) were particularly enriched in the type II neurons, whereas all SG neurons expressed the molecules essential for activating this cascade, despite the specific role of this signaling only in type I neurons. The striking aspect here is that autophagy is a regulator of *TGFβ* [23] and links Smad signaling [24]. Autophagy should regulate the function of SG neurons (type I and II) and play a key role for the neuronal development of SG neurons. A study [25] described that the autophagy protein *ATG7* is required for membrane trafficking and turnover in the axons, and impairment of axonal autophagy as a possible mechanism for axonopathy related to neurodegeneration. A recent study indicated that the initial stages of SGN and nerve fiber degeneration in the mouse cochlear cause the impairment of autophagy flux, while restoring autophagy–lysosomal pathway disruption by the translocation of TFEB (transcription factor EB) liking autophagy to lysosomal biogenesis into nuclear via inhibiting mTOR (mammalian target of rapamycin) cascade mitigated SGN and nerve fiber degradation [26]. These results suggested that the lysosome function via TFEB in autophagy–lysosome fusion step plays an essential role for restoring SGN and nerve fiber degradation.

## 4. Autophagy- and Lysosomal-Function-Related Genes and Hearing Loss

### 4.1. Autophagy-Related Genes Essential for Autophagosome Formation

#### 4.1.1. ATG5 Gene

As shown in Figure 1, the formation of the autophagosome requires the action of two evolutionarily-conserved ubiquitin-like conjugation systems (ATG5–ATG12 and LC3-PE), both of which require the *ATG5* gene [27,28]. *ATG5* is a key player for autophagic vesicle formation [29]. Knocking down in vitro or knocking out *ATG5* in vivo could result in downregulation or total inhibition of autophagy, suggesting that *ATG5* plays a central role in autophagy regulation. Thus, *ATG5* is one of the most commonly-targeted genes in autophagy gene-editing assays. *ATG5* also functions in the immune system, regulating innate and adaptive immune responses and is associated with autoimmune diseases, including SLE and autoinflammatory diseases, such as Crohn’s disease (Table 1). Inner-ear researchers [20] indicated that deletion of autophagy-related 5 (*ATG5*) resulted in hair cell (HC) degeneration and profound congenital hearing loss, generating mice deficient in *ATG5*. They indicated that both the morphology and mechanotransduction of *ATG5*-deficient auditory HCs were normal at P5, although polyubiquitinated proteins and p62 had already accumulated. However, at P14, polyubiquitinated protein aggregates and p62 progressively accumulated in auditory HCs of mice deficient in *ATG5,* as well as HC degeneration and profound hearing loss. They concluded that the cause of hearing loss in auditory HCs in mice deficient in *ATG5* is associated with degeneration of auditory HCs rather than maldevelopment. Hence, polyubiquitinated protein aggregates and p62 accumulation may play an important role in the progression of damage.

#### 4.1.2. miRNA 96 Gene

miRNA 96 is a member of the miRNA183 family (miRNA-183, miRNA-96, and miRNA-182) that is coordinately expressed from a single genetic locus in vertebrates. In the human genome, the miRNA-183 family cluster is located on chromosome 7q32 with a 4.5 kb region, including a locus that has been linked to autosomal-dominant non-syndromic hearing loss (NSHL) (*DFNA50*, OMIM #613074). As shown in Table 1, initially, two mutations in the seed region of miRNA-96 were detected in two Spanish families with autosomal-dominant progressive NSHL. Both mutations (+13 G > A and +14 C > A) affect the nucleolar targeting signals (NTSs) that are fully conserved among vertebrates (from fish to humans) and segregated with hearing loss in the affected families [30]. The description of novel causative variants within the *miRNA96* gene may be useful for clarifying the pathogenic mechanisms underlying the DFNA50-associated phenotype. Inner-ear researchers [31] detected the +57 T > C mutation as the third mutation of the *miRNA96* gene in humans that contributes quantitative defects in miRNA-96 related to the pathogenesis of sensorineural hearing loss, independent from additional qualitative defects (i.e., changes in the actual mature miRNA-96 sequence). The family carrying the +57 T > C mutation on hearing is characterized by late onset (between 25 and 40 years) and a slow progression of hearing impairment. Researchers indicated that autophagy is modulated dose-dependently by *miRNA96* through regulation of *mTOR* and *ATG7* required for the efficient formation of autophagosomes and suggested that the inhibition of mTOR by upregulation of *miRNA-96* may promote autophagy in prostate cancer, which is involved in maintaining a dynamic balance of *miRNA 96* in hypoxia [32]. Mutations of miRNA96 could make autophagy impaired through the activation of *mTOR* and the downregulation of *ATG7* in the cochlea, leading to sensorineural hearing loss.

### 4.2. Lysosomal-Function-Related Genes Essential for the Autophagy–Lysosome Pathway

The autophagy–lysosome pathway is an important mechanism for regulating the homeostasis of intracellular long-lived proteins and organelles [33,34]. Lysosomes release metabolites and ions that serve as a signaling hub for metabolic sensing and longevity, linking the functions of the lysosome to various pathways for intracellular metabolism and nutrient homeostasis [35]. The intraluminal pH of the lysosome is usually sustained in the low acidic range (4.2–5.3) for regulating many functions of lysosomes with the vacuolar-type ATPase (V-ATPase) acting as an ATP-dependent proton pump [36].

Based on these physiological characteristics, V-ATPases has been found to be deeply involved in the initiation of deafness [37]. In particular, dominant deafness-onychodystrophy (DDOD) syndrome caused by de novo mutation c.1516 C > N (p.Arg506X) in ATP6V1B2 is a rare disorder with chief complaints of severe deafness, onychodystrophy, and brachydactyly (Table 1) [38]. This group’s latest research [39] indicated four interesting results: (1) atp6v1b2 knockdown zebrafish had developmental defects in multiple organs and systems; (2) Atp6v1b2 c.1516 C > N knock-in mice led to cognitive disorders, based on the impaired hippocampal CA1 region from the pathology; (3) the normal hearing thresholds of Atp6v1b2 c.1516 C > N in 24-week-old knock-in mice, suggested that a compensation mechanism exists in the auditory system; and (4) V-ATPases assembly still occurred in Atp6v1b2 c.1516 C > N. However, the interaction between the E and B2 subunits was weaker than in the wild type (WT). They confirmed that the defectiveness of Atp6v1b2 leads to CNS impairments and extends the phenotype range of DDOD syndrome. ATP6V1B2 encodes the B2 subunit in V-ATPases, a multisubunit protein complex consisting of a soluble V1 subcomplex (responsible for hydrolyzing ATP) and a membrane-bound V0 subcomplex (involved in H+ translocation) expressed in almost all eukaryotes. Mutations in this gene theoretically result in lysosomal dysfunction or lysosomal damage. Consequently, autophagy dysfunction is caused by suppressing the degradation of autophagosomes in lysosomes, finally leading to cell death or aging. After this, these situations lead to distal renal tubular acidosis (dRTA, MIM: 602722), a rare disease characterized by metabolic acidosis and sensorineural hearing loss [40]. A recent genome-wide association study suggested that the ATP6V1B2 rs1106634 A allele increases the lifetime risk of depression and hippocampal cognitive deficits [41]. An abnormal rise in lysosomal pH, therefore, can have far-ranging effects on lysosomal digestion, strongly inhibiting hydrolases with the most acidic pH optima, but also potentially elevating activities of other hydrolases with pH optima closer to neutral. New reports implicate altered V-ATPase activity and lysosomal pH dysregulation in cellular aging [42], longevity [43], and adult-onset neurodegenerative diseases, including forms of Parkinson’s disease and Alzheimer’s disease [44]. Hence, the gene analysis of V-ATPase in the auditory–brain pathway may be a key to resolving the relationship between hearing loss and cognitive dysfunction.

Lysosomal storage diseases (LSDs) are inherited metabolic disorders caused by defects in lysosomal proteins or lysosomal-related proteins, which lead to lysosomal disfunction resulting in accumulation of undegraded substrate. LSD-associated genes encode different lysosomal proteins, including lysosomal enzymes and lysosomal membrane proteins [45]. Mutations in genes encoding lysosomal hydrolases, accessory proteins, membrane transport, or trafficking proteins may cause LSDs in vivo. LSDs are inherited in an autosomal recessive or, in some types, in an X-linked manner. As listed in Table 1, hearing loss has been found in several LSDs including Gaucher disease (caused by more than 400 mutations in the *GBA* gene (locus 1q21), encoding for the lysoglucosylceramide-degrading enzyme β-glucocerebrosidase (EC 3.2.1.45)) [46], Fabry disease (X-linked glycosphingolipidosis caused by deficiency of the lysosomal α-galactosidase A (EC 3.2.1.22), encoded by the *GLA* gene (Xq22.1)) [47], Pompe disease (a deficiency in the lysosomal α-glucosidase (EC 3.2.1.3) encoded by the *GAA* gene (17q25.3)) [48], Niemann–Pick type C (NPC) (mutations in *NPC1* (18q11.2) and *NPC2* (14q24.3) genes, intralysosomal cholesterol, and sphingolipid accumulation) [49], and mucopolysaccharidoses (mutations in the *IDUA* gene providing instructions for producing an enzyme (α-L-iduronidase), which is involved in the breakdown of glycosaminoglycans (GAGs)) [50]. LSDs are caused by disruptions in the lysosomal network and intralysosomal accumulation of substrates in certain cell types. However, many aspects of the molecular pathology of the cochlea due to LSDs remain unclear. According to the recent study [51], it will be interesting to shed light on whether the endosomal sorting complex required for transport (ESCRT)-dependent membrane sealing is involved in mammalian LSDs caused by different genetic defects and whether lysophagy—one of selective autophagy and ESCRT repair—acts in concert during the development of lysosomal storage [52].

## 5. Congenital Disorder of Autophagy with Hearing Loss

### β-Propeller Protein-Associated Neurodegeneration (BPAN): Mutations in the WDR45 Gene

Some congenital disorders of autophagy with an emerging phenotype of inborn errors of metabolism involve hearing impairment as one of the associated symptoms (Table 1) [53]. Recently, two groups independently reported mutations in the *WDR45* gene as the genetic cause of β-propeller protein-associated neurodegeneration (BPAN), a disease that had been previously labeled using the term ‘static encephalopathy of childhood with neurodegeneration in adulthood (SENDA) syndrome’ [54]. This disease is characterized by the onset of dystonia, Parkinsonism, and progressive cognitive decline with visual and auditory disabilities in early adulthood or adolescence. *WDR45*, also known as *WIPI4*, is located on the X-chromosome and is one of the four mammalian homologs of the core autophagy gene *epg-6* in *Caenorhabditis elegans* [55]. *WDR45* encodes a WD repeat protein, a superfamily of proteins with a conserved core of 40 amino acids terminating in tryptophan–aspartic acid (WD) residues. WD40 proteins fold into similar β-propeller structures that function as protein–protein autophagy or protein–DNA interaction platforms and mediate molecular signaling cascades mainly through the smaller top surface [56]. Based on these properties, WD-repeat proteins consist of components with many essential biologic functions and pathways including autophagy. Importantly, *WDR45*, the WD-repeat protein mutated in BPAN, interacts with autophagy-related proteins *ATG2* and *ATG9* to regulate crucial steps for autophagosome formation and elongation [57]. Therefore, depletion of *WDR45* in mammalian cells could lead to the accumulation of early autophagic vesicles or immature autophagosomes [58]. According to a recent report [59], conditional CNS-specific *WDR45* knockout mice (Nes-WDR45fl/Y) show swollen axons and accumulation of autophagy substrates p62 and ubiquitin as the characteristics of axonal pathology. Neither neurodegeneration nor iron deposition are prominent phenotypes. However, at the behavioral level, Nes-WDR45fl/Y mice displayed subtle deficits of coordinated motor skills, poor memory, and learning impairment. These situations indicated deficits in neuronal circuit formation or neurotransmission. Another aspect of the pathogenesis of BPAN should be the role of autophagy in iron metabolism, which is called ferritinophagy as selective autophagy. The bioavailability of intracellular iron is critically regulated by the delivery of ferritin to autophagosomes and the degradation in lysosomes, allowing release of iron into the cytoplasm [60,61]. These recent studies showed that hearing impairment in BPAN may be related to the accumulation of p62 and ubiquitin in neural cells of central auditory pathway or ferritinophagy impairment.

## 6. The Effect of Autophagy for Genetics of Hearing Loss

### 6.1. Genetics of Sensorineural Hearing Loss (DFNA5 and DFNB59) and Autophagy

*DFNA5* was first identified in a Dutch family as a gene causing autosomal dominant hearing loss (HL). In almost all cases, the *DFNA5* mRNA transcript skips exon 8, leading to a frameshift and a premature truncation of the protein [62]. DFNA5-associated HL is characterized by non-syndromic HL with no other symptoms. A recent study [63] indicated that gasdermin-E (GSDME), which was originally identified as *DFNA5* (deafness gene, autosomal dominant 5) [64], could transform caspase-3-mediated apoptosis induced by chemotherapy drugs, etc., into pyroptosis, an inflammatory form of programmed cell death. GSDME was specifically cleaved by caspase-3-mediating cleavage of autophagy-associated protein beclin-1, inactivating autophagy and promoting apoptosis. Beclin-1 is a dual regulator for both autophagy and apoptosis and a substrate of caspase-3 with two cleavage sites at positions 124 and 149 [65]. The N-terminal domain of GSDME, as the functional characteristic, displays an apoptosis-inducing activity while the C-terminal domain functions as an apoptosis-inhibiting regulator by shielding the N-terminal domain [66]. A specific form of autosomal dominant progressive sensorineural hearing loss due to DFNA5 may cause the disruption of balance among apoptosis, pyroptosis, and autophagy in sensory hair cells.

**Figure 2:**
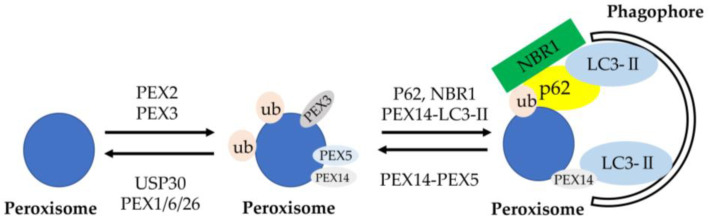
Schematic of molecular mechanisms of pexophagy. Peroxisome membrane proteins are ubiquitinated by the E3 ubiquitin ligase, *PEX2*, to designate peroxisomes for pexophagy. Opposing the action of PEX2 on peroxisomes is the deubiquitinating enzyme USP30. Ubiquitinated peroxisome membrane proteins are removed from peroxisomes by the AAA-type ATPase (*PEX1–PEX6–PEX26*) and the deubiquitinase USP30 to prevent pexophagy. Increasing the expression of *PEX3* on peroxisome membranes may also designate them for pexophagy. Ubiquitinated peroxisomes are targeted to autophagosomes through interactions with the autophagy receptors NBR1 and p62, which facilitate sequestration within autophagosomes through binding with LC3-II. Peroxisomes are also targeted and sequestered within autophagosomes when LC3-II out-competes *PEX5* for binding to *PEX14*. Import-competent peroxisomes deter pexophagy through *PEX14–PEX5*-binding, whereas import-incompetency frees *PEX14* allowing it to bind LC3-II and facilitate pexophagy [71].

*DFNB59* was the first reported human gene leading to nonsyndromic deafness due to neuronal defect through the auditory pathway neurons [67]. Nonsense mutations in the *PJVK* gene encoding protein PJVK, which is present in hair cells supporting cells and spiral ganglion cells, resulted in autosomal recessive nonsyndromic deafness in humans at the DFNB59 locus on chromosome 2q31.2 [68,69]. A recent study indicated that the *DFNB59* form of deafness is a pexophagy disorder [70]. Pexophagy means that peroxisomes are degraded by lysosomes through autophagic pathways as a selective autophagy (Figure 2) [71]. Peroxisome membrane proteins are ubiquitinated by PEX2, the E3 ubiquitin ligase, for inducing pexophagy. Ubiquitinated peroxisome membrane proteins are removed from peroxisomes by the AAA-type ATPase PEX1–PEX6–PEX26 and the deubiquitinase USP30 for preventing pexophagy. The expression of PEX3 on peroxisome membranes also increases for inducing pexophagy. Ubiquitinated peroxisomes are bound to autophagosomes through interacting with the autophagic adapter proteins (cargo receptors), NBR1 and p62, and facilitating its binding to LC3-II. Peroxisomes are also sequestered into autophagosomes when PEX14 interacts with LC3-II rather than PEX5. Peroxisomes are dynamic organelles whose metabolism, size, abundance, and phenotype can change in response to alterations in nutritional and other environmental conditions. Peroxisomes are routinely turned over by pexophagy for the quality control of organelles, referred to as peroxisome dynamics, for several processes of peroxisome biogenesis. *PJVK* also has another function—triggering pyroptosis when pexophagy is induced by oxidative stress [72,73,74,75]. DFNB59 could play an essential role in oxidative-stress-induced peroxisome biogenesis and pexophagy in auditory hair cells [70]. Hence, autosomal-recessive non-syndromic hearing loss caused by *DFNB59* mutations may be affected by the impairment of pexophagy in sensory hair cells in terms of progressive hearing loss.

### 6.2. Presbycusis Accelerated by Connexin 26 Partial Loss and Autophagy through Nrf2/Keap1 Pathway

**Figure 3:**
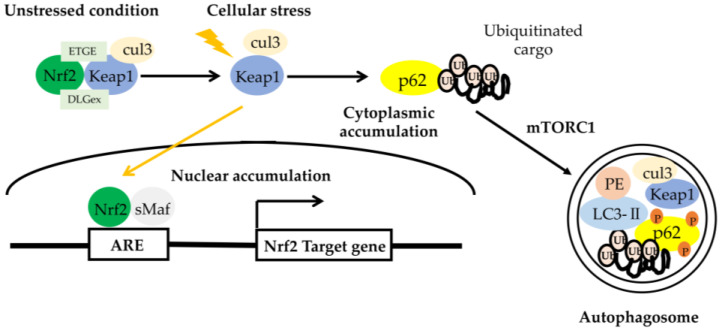
The role of the p62– Kelch-like ECH-associated protein 1 (Keap1)–nuclear factor (erythroid-derived-2)-like 2 (Nrf2) axis. Upon selective autophagy, oligomerized p62 undergoes phosphorylation at Ser residues (S407, S403) (shown as ‘P’) and increases the binding affinity of p62 to ubiquitin, followed by sequestration of polyubiquitinated cargos. Then, mTORC1 phosphorylates S349 of p62 and increases the binding affinity of p62 to Keap1, resulting in the escape of Nrf2 from the Keap1 interaction. Free Nrf2 enables the activation of various target genes. Keap1 is degraded together with the polyubiquitinated cargo-binding to p62 into autophagosome. ARE, antioxidant response element [82].

Mutations in the gap junction protein β-2 (*GJB2*) gene encoding connexin 26 (*Cx26*) are the most common cause of sensorineural hearing impairment [76,77,78,79]. In several populations, the truncating variant 35delG involved in the prevalent *GJB2* mutation results in a complete loss of function of *Cx26* protein whose structure has been solved with a 3.5 Å resolution. A recent study indicated that the partial loss of *Cx26* results in accelerated presbycusis (age-related hearing loss (ARHL)) caused by redox imbalance and dysregulation of the nuclear factor (erythroid-derived-2)-like 2 (Nrf2) pathway [80]. It was confirmed that the hearing level more rapidly worsened in *Gjb2*+/− mice than control mice using auditory brainstem responses (ABRs) and distortion product otoacoustic emission (DPOAE) thresholds. Levels of oxidative stress increased in the cochlear duct of the auditory phenotype of *Gjb2*+/− mice and, as a result, apoptosis was induced, the release of glutathione from connexin hemichannels was reduced, nutrient delivery to the sensory epithelium via cochlear gap junctions was decreased, and the expression of target genes of Nrf2 was deregulated. Conversely, *Gjb2*−/− mice failed to express acquired deafness although levels of oxidative stress increased in the cochlea [81]. This research group also indicated that two NRF2 target genes (*PRKCE* and *TGFβ1*) (*p*-value < 4 × 10^−2^) were detected in a large cohort of 4091 individuals with hearing phenotype (including 1076 presbycusis patients and 1290 healthy matched controls from Europe, Caucasus, and Central Asia) by a genome-wide association study. In this study, the authors suggested from the both basic research and clinical study that: (1) it is important for hearing maintenance to normally run the Nrf2 pathway and (2) dysfunction of the Nrf2 pathway may result in human presbycusis. As shown in Figure 3, Nrf2 is a transcription factor in response to gene expression of antioxidant proteins [82]. The common Nrf2-binding motif known as the antioxidant response element (ARE) should be activated for inducing Nrf2 target genes [83]. In auditory cells under oxidative stress, an autophagic pathway is maintained by a Kelch-like ECH-associated protein 1 (Keap1)–Nrf2 feedback loop through p62, a protein encoded by the sequestosome 1 gene (SQSTM1) [84]. Kelch-like ECH-associated protein 1 (Keap1) is an adaptor protein of cullin-3-based ubiquitin ligase. The N-terminally lying Neh2 domain of Nrf2 contains two Keap1-binding motifs, DLG and ETGE. Interactions between these two binding motifs and Keap1 compose a key regulatory site for Nrf2 activity through the formation of a two-site-binding hinge-and-latch mechanism, although this two-site binding is necessary for ubiquitinated Nrf2 [85,86]. ETGE tightly binds to Keap1, whereas DLGex binds more weakly than ETGE. Here, this binding plays a role as a fine-tuner of the ubiquitination of Nrf2 [87]. Nrf2-repressor function was lost by chemical modification of specific cysteine sensors of KEAP1 by oxidative stressor, and then Nrf2 was released from the Keap1 interaction and translocated into the nucleus to induce the expression of Nrf2-target gene [88,89]. The Keap1–Nrf2 system functions as a major oxidative stress response pathway in auditory cells. *p62/SQSTM1* is a stress-inducible protein with multifunctional domains including an LC3-interacting region (LIR), a Keap1-interacting region (KIR), and a ubiquitin-associated (UBA) domain [90], regulating the activation and stabilization of Nrf2 by inhibiting the ability of Keap1 to hold Nrf2. It also functions as an adaptor protein between selective autophagy and ubiquitin signaling [91,92]. This means that the Keap1–Nrf2 pathway and selective autophagy could be mediated by *p62/SQSTM1*. Taken together, p62-mediated selective autophagy may regulate presbycusis accelerated by *Cx26* partial loss through Nrf2/Keap1 pathway in cochlea.

## 7. Conclusions

**Figure 4:**
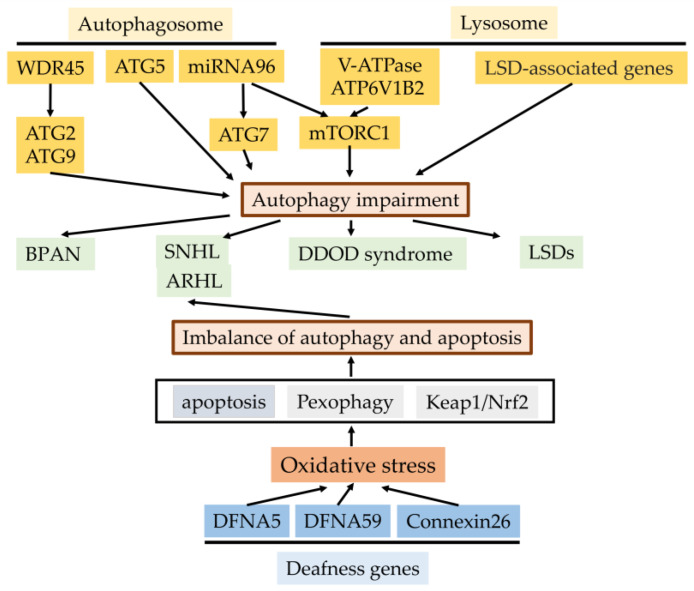
The effects of the autophagy process for genetics of hearing impairment and autophagy- and lysosomal-function-related genes for hearing impairment. Genetic-defect-linked autophagy- and lysosomal-related genes, including mutation or deletion result in sensorineural hearing loss or hereditary disorders with sensorineural hearing loss (BRAN, DDOD syndrome, and LSDs). Mutations of three deafness genes (DFNA5, DFNA59, and connexin26) linked with autophagy induce oxidative stress in the cochlea and the resulting imbalance of autophagy and apoptosis in sensory hair cells due to depressed pexophagy or Keap1/Nrf2, causing the progression of SNHL or ARHL (Cx26 partial loss). BRAN, β-propeller protein-associated neurodegeneration; SNHL, sensorineural hearing loss; ARHL, age-related hearing loss; DDOD syndrome, dominant deafness–onychodystrophy; LSDS, lysosomal storage diseases.

In this review article, we summarized the effects of the autophagy process on the genetics of hearing impairment and autophagy–lysosomal function-related genes for hearing impairment (Figure 4). Sensorineural hearing loss (SNHL) may be caused by both environmental and hereditary factors. Approximately 60% of cases are due to genetics. We described how three important deafness genes (*DFNA5, DFNA59* and *connexin26*) linked with autophagy are sensitive to oxidative stress, inducing SNHL, or age-related hearing loss (ARHL), and that autophagy deficiency caused by autophagy- and lysosomal-function-related genes can induce hearing impairment. Taken together, autophagy may play crucial roles in the genetics of hearing loss. However, this remains speculative, as few genes related to the autophagy process as a gene-causing autosomal dominant hearing loss have been detected to date. Exploring genes related to the autophagy–lysosome pathway will provide new insight into the genetics of hearing impairment in the near future. In conclusion, investigating the autophagy–lysosomal-function-related genes will open new doors for the therapeutic targets of sensorineural hearing loss. Furthermore, we hope that in the near future, new investigations into the genetic variants of autophagy- and lysosomal-function-related genes will be conducted based on the American College of Medical Genetics (ACMG) guidelines.

## Figures and Tables

**Table 1:** Characteristics of autophagy- and lysosome- related genes inducing sensorineural hearing loss.

Gene	Gene Locus	Encoding	Genetic Defects	Related Disease	Affected Process of Autophagy
*Atg5*	6q21	ATG protein	Deletion	Autoinflammatory disease Autoimmune disease	Autophagosome formation
*miRNA96*	7q32.2	DFNA50 (OMIM #613074)	Point mutations	Sensorineural hearing loss	Autophagosome formation
*WDR45*	Xp11.23	WD repeat protein	Uncovered mutations	BPAN	Autophagosome formation
*GBA*	1q21	(Lyso)glucosylceramide	Missense mutations Point mutations Deletions Insertions Splicing aberrations Various rearrangements	Gaucher disease Type 1 (GD1) Type 2 (GD2) Type 3 (GD3)	Lysosome biogenesis
*GLA*	Xq22.1	lysosomal α-galactosidase A	Missense mutations Nonsense mutations Splicing mutations Deletions Insertions	Fabry disease	Lysosome biogenesis
*GAA*	17q25.3	lysosomal α-glucosidase	Nonsense mutations Multiple exon deletion	Pompe disease	Lysosome biogenesis
*NPC1*	18q11.2	NPC protein	Missense mutations Point mutation Duplication mutation Splicing mutation Frame deletion	Niemann–Pick type C	Lysosome biogenesis
*NPC2*	14q24.3	NPC protein	Missense mutations of homozygous state	Niemann–Pick type C	Lysosome biogenesis
*IDUA*	4p16.3	alpha-L-iduronidase	Missense mutations Nonsense mutation Deletion	Mucopolysaccharidoses	Lysosome biogenesis

WD repeat: tryptophan-aspartic acid (WD) residues; BRAN: Beta-propeller protein-associated neurodegeneration; DDOD: Dominant deafness-onychodystrophy.
